# X-Linked Muscular Dystrophy in a Cat with a Putative Variant in the *DMD* Gene

**DOI:** 10.3390/ani16081278

**Published:** 2026-04-21

**Authors:** Harry Cridge, Caylen Erger, Kyan Thelen Strong, Ling T. Guo, Hong An, Chunhui Xu, G. Diane Shelton

**Affiliations:** 1 Department of Small Animal Clinical Sciences, College of Veterinary Medicine Michigan State University, East Lansing, MI 48823, USA; 2 Comparative Neuromuscular Laboratory, Department of Pathology, School of Medicine, University of California San Diego, LaJolla, CA 92093, USA; 3 Bioinformatics and Analytics Core, University of Missouri-Colombia, Colombia, MO 65211, USA

**Keywords:** muscle, myopathy, dystrophin, feline, *CLIC2*

## Abstract

X-linked dystrophin-deficient muscular dystrophy (DD-MD) is an uncommon neuromuscular disorder in cats. We described an adult male cat with chronic tongue protrusion, dysphagia, muscle hypertrophy, and a history of rhabdomyolysis associated with anesthesia. Clinical pathology revealed markedly increased CK activity, muscle histopathology demonstrated a dystrophic phenotype, and an absence of dystrophin protein was confirmed by immunofluorescent staining. Whole genome sequencing identified two potential disease-causing variants, including a new missense variant in the *DMD* gene (c.2207T>C; p.Gln736Arg), which was considered causative of the clinical phenotype. A second variant in the *CLIC2* gene was also detected but was considered unlikely to cause myopathic signs. The clinical course remained stable over 1.5 years with supportive management and dietary modification, and no further episodes of rhabdomyolysis occurred. This case expands the known spectrum of feline *DMD* variants and highlights the value of genetic testing combined with muscle histopathology for diagnosing chronic presentations of MD. Avoidance of inhalant anesthetics may be important in managing affected cats due to the risk of acute muscle injury.

## Simple Summary

Muscular dystrophies are a heterogenous group of rare inherited disorders that, in some forms, including dystrophin deficiency, can lead to muscle hypertrophy and atrophy, dysphagia, and gait abnormalities. Here, we report a rescued adult male DSH cat with a chronic history of an enlarged tongue, difficulty swallowing, increased respiratory effort, and an abnormal gait. The cat also developed rhabdomyolysis following an episode of anesthesia. Creatine kinase (CK) activity was markedly and persistently elevated, and muscle histopathology showed a dystrophic phenotype. Dystrophin deficiency was confirmed with immunofluorescent staining of muscle cryosections using dystrophy associated antibodies. Whole genome sequencing identified a previously unknown variant in the *DMD* gene (VarElect Score: 183.84) that was likely causative. An additional variant in the *CLIC2* gene (VarElect Score: 3.26) was detected, but this variant was unlikely to cause the cat’s clinical phenotype. While there is no known cure for any type of muscular dystrophy, supportive care and avoidance of anesthesia may help in improving day-to-day quality of life.

## 1. Introduction

Dystrophin, encoded by the *DMD* gene, is essential for maintaining muscle fiber integrity by linking the intracellular cytoskeleton to the extracellular matrix via the dystrophin-associated glycoprotein complex (DAGC). Disruption of the protein compromises sarcolemmal stability during muscle contraction, leading to progressive myofiber damage. X-linked dystrophin-deficient muscular dystrophy (DD-MD) is an uncommon neuromuscular disease in cats, and is characterized by symmetrical skeletal muscle hypertrophy, dyspnea, variable tongue hypertrophy leading to dysphagia, and an abnormal gait [1,2,3,4,5,6,7,8,9,10,11,12,13]. In recent years, expanded availability of next-generation sequencing has allowed for the identification of four pathologic variants and their correlation with variable clinical phenotypes. Mild clinical phenotypes and a later onset of disease have been reported in a domestic cat with a missense *DMD* variant (c.4186C>T (p.His1396Tyr)) and a domestic shorthair cat with a nonsense *DMD* variant (c.8333G>A (p.Trp2778Ter)) [11,13]. Severe clinical phenotypes with early onset of clinical signs have been reported in a domestic shorthair cat with a nonsense *DMD* variant (c.4849C>T (p.Gln1617Ter) and a Maine Coon cat with a nonsense *DMD* variant (c.4186C>T (p.His1396Tyr) [10,12]. In this study, we reported a new missense variant in the *DMD* gene (c.2207T>C (p.Gln736Arg) in an adult male cat with DD-MD.

Two forms of DD-MD are reported in humans, each with a different clinical course and prognosis. Duchene-type muscular dystrophy is a rapidly progressive early-onset disease with a poor prognosis [14,15]. In contrast, Becker muscular dystrophy is a disease which typically has a later onset of clinical signs, slower rate of progression, and variable clinical severities [16,17]. In cats, the clinical presentation of DD-MD may mimic that of the above syndromes, with some cats having juvenile-onset of severe clinical signs, while others have a delayed onset with a more subdued clinical course [10,11].

## 2. Case Summary

An approximately 6-year-old male neutered domestic shorthair cat presented to the Michigan State University Veterinary Teaching Hospital (MSU-VTH) for evaluation of progressive non-productive ‘retching’, potential dysphagia, and tachypnea. No other clinical signs were noted. The cat was adopted 2 years prior to evaluation and was reported to maintain an open mouth posture with tongue protrusion, and a preference to lick rather than chew food since adoption.

Approximately 4 months after adoption the cat underwent a dental procedure at the primary care veterinarian due to the presence of moderate dental tartar and gingivitis. Pre-operative bloodwork was reportedly normal with the exception of a mildly increased ALT activity (183 U/L, RI: not reported). CK activity was not measured. Anesthetic pre-medications included buprenorphine followed by butorphanol, ketamine, and dexmedetomidine. Isoflurane inhalant anesthesia was used during the procedure. Four teeth were extracted, and during recovery, the cat demonstrated open mouth breathing, decreased Sp02 (92%), and generalized discomfort. The patient was subsequently provided oxygen, furosemide (2 mg/kg IV), heparin (200 IU/cat), and dexamethasone (0.2 mg/kg IV) and was discharged home. The cat did poorly at home, characterized by increased respiratory effort, vocalization, discomfort, and an elevated respiratory rate, and was subsequently presented to an emergency facility for evaluation. Evaluation at the emergency facility revealed normal vital parameters, a normal left atrium-to-aorta ratio on point-of-care ultrasound, increased respiratory effort and no evidence of a murmur or arrythmia. The cat was noted to be reluctant to utilize the limbs but was ambulatory when encouraged.

CBC revealed no clinically significant abnormalities with the exception of mild thrombocytosis (571,000/µL, RI: 168–438 k/µL) and mild eosinophilia (2300/µL RI: 0.1–1.9 k/µL). Serum chemistry revealed azotemia (BUN 64 mg/dL, RI: 19–36, Crea: 2.5 mg/dL, RI: 1.0–2.3) with a concurrent USG of 1.015. Serum chemistry also revealed hyperphosphatemia (16.4 mg/dL, RI: 2.7–5.7), hyponatremia (Na: 140 mmol/L, RI: 145–155), hypochloridemia (94 mmol/L, RI: 110–123 mmol/L), hypocalcemia (iCa: 3.1 mg/dL, RI: 5.1–6.0), and hyperglycemia (408 mg/dL, RI: 78–143). No glucosuria was evident. The cat also demonstrated markedly elevated CK activity (1,879,920 U/L, RI: 46–490), with corresponding moderately elevated AST (4088 U/L, RI: 14–36) and ALT activities (424 U/L, RI: 25–76). Serum toxoplasma gondii titers were negative (IgM and IgG). A PTH profile revealed a physiologically appropriate increase in PTH concentration (103.9 pmol/L, RI: 0.70–3.40). Thoracic radiographs were performed due to the respiratory signs and revealed an unstructured interstitial pulmonary pattern in the caudal lung lobe. There was no evidence of cardiomegaly or cardiac decompensation, although the animal had recently received furosemide.

The cat was provided with ampicillin sulbactam (30 mg/kg IV q 8 hr) in case of an aspiration event under anesthesia. The cat was also prescribed calcium gluconate, IV fluid therapy guided by urinary output, and methadone (0.1 mg/kg IV q 6 hr). Presumed rhabdomyolysis was diagnosed and the cat was discharged after 7 days of supportive and symptomatic care. Recheck examination performed 11 days later revealed clinical improvement, the CK activity had improved to 2704 U/L (RI: 46–490 U/L), and the hypocalcemia (tCa: 10.9 mg/dL, RI: 9.1–10.7) resolved. Around 1 year after the presumed rhabdomyolysis event the cat presented to the primary veterinarian for intermittently hard stools and constipation, which were managed with MiraLAX (1/8 tsp as needed). The owner reported that the cat would cough while eating.

**Figure 1:**
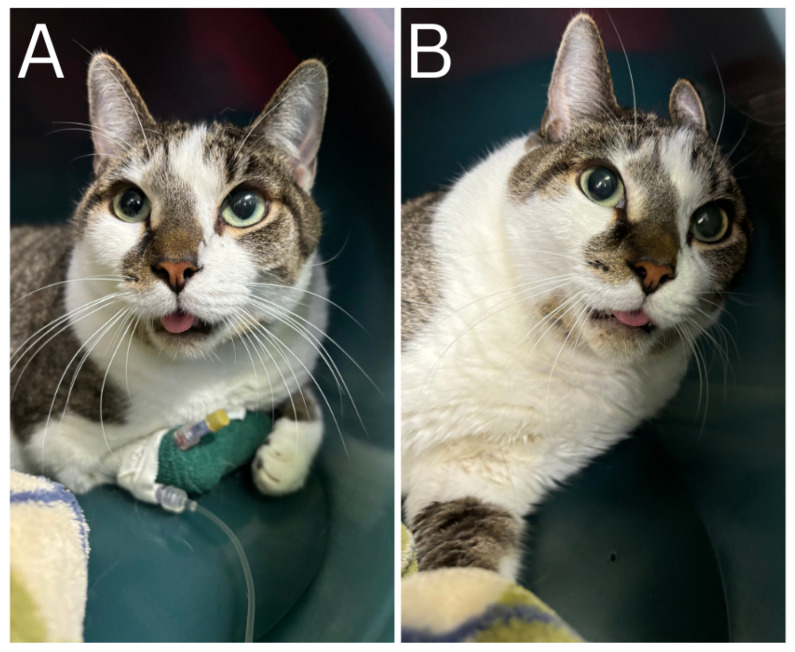
Six-year-old male neutered cat with a protruding tongue (**A**) and prominent cervical musculature (**B**).

During presentation to MSU-VTH for non-productive ‘retching’, potential dysphagia, and dyspnea, the cat was noted to have prominent cervical musculature, a protruding tongue (Figure 1), and increased respiratory effort. The remainder of the examination was normal, with the exception of an elevated respiratory rate (60 bpm). On additional questioning the owner reported intermittent hypersalivation and anorexia, long-standing tongue protrusion and no recent changes in the cervical musculature. The owner also reported that the cat has always had an unusual gait.

A minimum database was performed. The CBC revealed no significant abnormalities. The serum chemistry revealed mild hyperglycemia (177 mg/dL, RI: 78–143) in addition to evidence of muscle injury (CK activity: 125,187 U/L, RI: 46–490, AST activity: 777 U/L, RI: 14–36, and ALT activity 150 U/L, RI: 25–76). Thoracic radiographs revealed mild generalized cardiomegaly without vascular congestion, and an echocardiogram was not pursued. A mild bronchial pulmonary pattern was also noted diffusely throughout the lungs. An abdominal ultrasound was performed, which revealed diffuse muscularis thickening of the duodenum and jejunum along with a hyperechoic mucosal layer. Bilateral renal degenerative changes and cystic changes to the medial iliac lymph nodes measuring 2.0 × 2.2 cm and 2.7 × 1.8 cm were identified. Ultrasound-guided lymph node aspirates under sedation revealed mixed eosinophilic, macrophagic, and neutrophilic inflammation, and lymph node aspirate culture was negative. Aspirates of the liver and spleen revealed no abnormalities. Repeat serum toxoplasma titers remained negative. It was discussed that the non-productive retching could be respiratory or gastrointestinal in origin, and biopsies of the gastrointestinal tract, medial iliac lymph nodes, and forelimb musculature were offered and pursued by the client. The cat was pre-medicated with butorphanol (0.2 mg/kg IV), followed by an alfaxalone induction and maintenance to effect. Inhalant anesthesia was avoided. Recovery from anesthesia was uneventful with no episodes of dysphoria or discomfort. While awaiting the results of the biopsies the cat was discharged on gabapentin (10 mg/kg PO q 8 hr) for analgesia, maropitant citrate (1.6 mg/kg PO q 24 h) and ondansetron (0.4 mg/kg PO q 8 hr) for anti-emetic effect, and mirtazapine for appetite stimulation (0.38 mg/kg PO q 24 h).

Histopathology of the duodenum, jejunum, and ileum revealed a mild lymphoplasmacytic to eosinophilic enteritis, and the lymph nodes revealed chronic cystic dilation consistent with potential nearby lymphatic obstruction. Lymph node tissue culture was negative. The cat was initially transitioned to a hydrolyzed diet (Hills Z/D dry), but due to persistent clinical signs of ‘retching’ and ptyalism, the cat was placed on a novel protein diet (Purina Sensitive Stomach Fish, canned) which improved the clinical signs. The cat was noted to tolerate the canned diet much better than dry kibble. It was suspected that the cat’s episodes of ‘retching’ were secondary to the underlying food-responsive enteropathy and/or secondary to a dysphagia related to the tongue hypertrophy.

**Figure 2:**
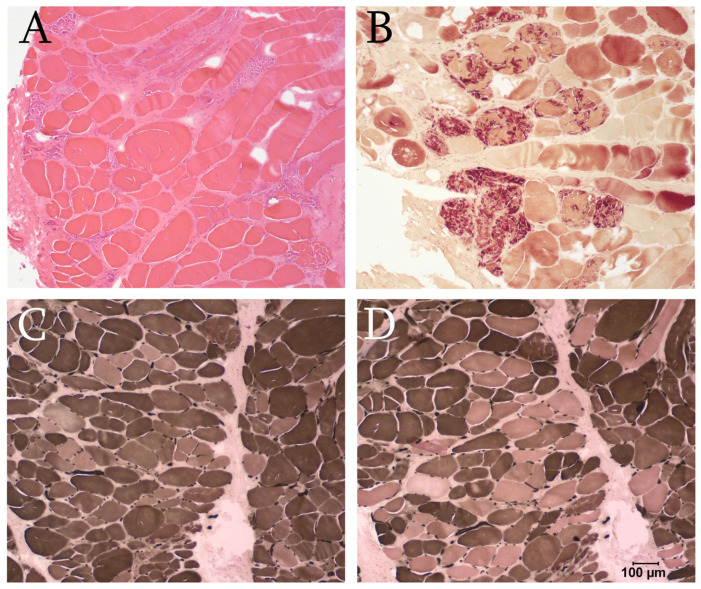
(**A**) Representative cryosection from the triceps muscle showing degeneration in multifocal areas (H&E stain). (**B**) Multiple necrotic (degenerating) fibers undergoing phagocytosis were identified (esterase reaction). (**C**,**D**) An increased population of type 2C fibers indicative of regeneration is illustrated by the ATPase reaction for fiber typing (ATPase reaction pH 9.8 (**C**) and ATPase reaction pH 4.3 (**D**) are shown. At pH 9.8, type 1 fibers have light staining and type 2 fibers have dark staining. At pH 4.3, type 1 fibers have dark staining, type 2 fibers have light staining and type 2C (regenerating) have tan staining. Bar in lower right image = 100 µm for all images.

Unfixed chilled and fixed biopsies were collected from the triceps and sternocephalicus muscles then shipped by a courier service under refrigeration to the Comparative Neuromuscular Laboratory, University of California San Diego. Following freezing in isopentane pre-cooled in liquid nitrogen, cryosections were cut and evaluated in 8 µm sections using a standard panel of histochemical stains and reactions, and the fixed samples were evaluated in routine paraffin sections [18]. A degenerative and regenerative myopathy consistent with a dystrophic phenotype was found in both muscles (Figure 2).

**Figure 3:**
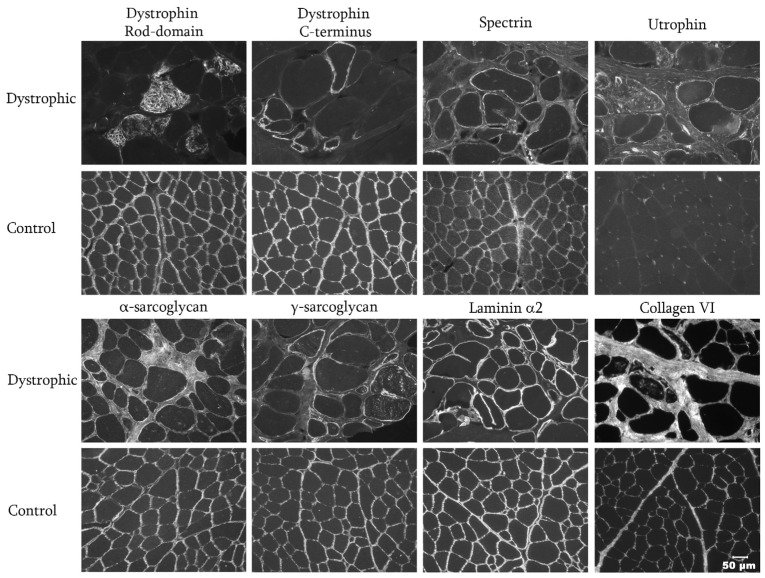
Immunofluorescent staining of cryosections from the triceps muscle of the affected cat and an archived control muscle for identification of the presence or absence of muscular dystrophy-associated proteins. Sarcolemmal staining for the rod domain and carboxy terminus of dystrophin was absent compared to the control muscle with rare revertant fibers showing mislocalized internal staining. Utrophin staining was increased compared to the control as typically found in dystrophin deficiency. Staining for other dystrophy-associated protein was similar to the control muscle. Collagen VI staining was increased, supporting endomysial and perimysial fibrosis. Bar in lower right corner equals 50 µm for all images.

To further characterize a specific form of disease, cryosections from the triceps muscle of the affected cat and an archived control muscle were cut (8 μm) and stained for indirect immunofluorescence as previously described (Figure 3) [19]. Several monoclonal or polyclonal antibodies were used, including those against the rod (1:100, NCL-DYS1) and carboxy-terminus (1:100, NCL-DYS2) of dystrophin, against utrophin (1:20, NCL-DRP2), α and γ-sarcoglycan (1:200, gift of Eva Engvall) [20], laminin α2 (gift of Eva Engvall, 4F11, direct apply) [21] and collagen VI (gift of Eva Engvall, 3G7, direct apply) [22]. Sarcolemmal staining for the rod domain of dystrophin was absent and patchy with the revertant fibers showing internalized staining using the antibody against the carboxy terminus. Staining for spectrin was similar to the control, indicating good tissue quality. Staining intensity for utrophin was increased on both regenerating and non-regenerating fibers. Staining for α and γ-sarcoglycan and laminin α2 was similar to the control. The staining pattern for collagen VI was increased, indicative of endomysial and perimysial fibrosis. DD-MD was diagnosed.

## 3. Whole Genome Sequencing and Candidate Gene Analysis

**Figure 4:**
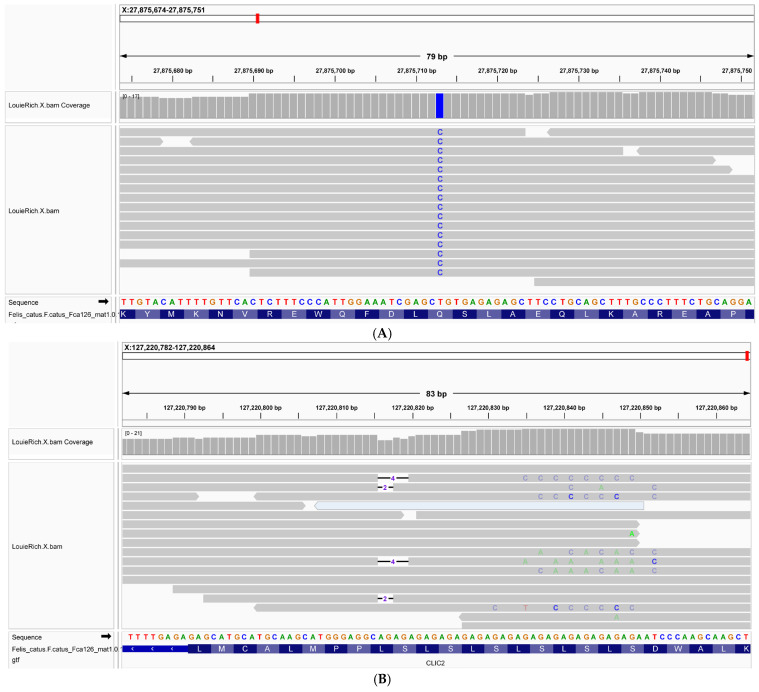
(**A**) shows sequencing coverage at the site of a missense variant in the *DMD* gene (c.2270T>C; p.Gln736Arg) from a single affected cat. Multiple independent sequencing reads cover this position, and all reads demonstrate the same nucleotide change from the reference-based T to C. There is no evidence of the reference allele among the reads, indicating that both copies of the gene carry the variant. This consistent finding across all reads supports a homozygous mutation at this site. (**B**) shows sequencing coverage at the site of the frameshift variant in the *CLIC2* gene (c.82330823CAC>C (p.Leu275Ter)). Multiple independent sequencing reads cover this position. Some of the reads show a 2 or 4 basepair deletion that generates the frameshift variant.

Given the diagnosis, EDTA whole blood was collected for whole genome sequencing (WGS). Whole blood was stored at −80 °C until DNA was extracted using the DNeasy ^®^ Blood & Tissue Kit (Qiagen, Hilden, Germany) following the manufacturer’s instructions. Extracted DNA was then submitted to the University of Missouri Genomics Technology Core (Columbia, MO, USA) for WGS. The raw reads were trimmed and filtered using fastp with its default parameters to obtain the clean reads [23]. Variant calling was performed with a custom Nextflow workflow to highly parallelize the processes [24]. Clean reads were mapped to the cat reference genome F.catus_Fca126_mat1.0 using Minimap 2 [25]. Next, duplicated reads were marked and removed using MarkDuplicates in GATK (v4.2.6.1), and variants for each sample were called using HaplotypeCaller in GATK (v4.2.6.1) [26]. Finally, hard filters for both SNPs and InDels were applied with VariantFiltration in GATK (v4.2.6.1) following GATK best practices [27], and all the variants were further annotated with Variant Effect Predictor (VEP) and VarElect [28,29]. Variants were confirmed by visual inspection using the Integrative Genomics Viewer (Figure 4) [30]. All identified variants are available in Supplementary Material S1. Amino acid protein domain annotation was performed using a script which utilized InterProScan (v5.75-106.0) Linux version (v5.75-106.0) [31]. The feline DMD protein sequence, ENSFCTP00005038844; DMD-205, was used as input in FASTA format. InterProScan was run with default parameters, enabling domain prediction across multiple integrated databases, including SMART [32], Pfam [33], Gene3D [34], and SUPERFAMILY [35]. Additional options were enabled to retrieve Gene Ontology (GO) terms and pathway annotations.

**Figure 5:**
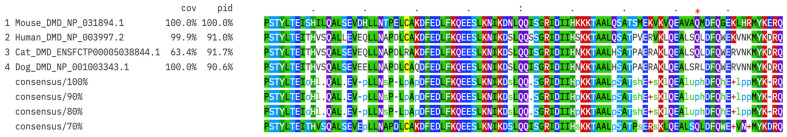
Multiple sequence alignment revealed that the residue corresponding to the feline variant site is highly conserved across species. The red star indicates position 736, where the glutamine (Q) is observed in humans, mice, and cats, while a conservative substitution to arginine (R) is present in dogs. Both glutamine and arginine are polar residues capable of participating in hydrogen bonding, suggesting that physicochemical properties at this site are maintained across species.

One high-impact (frameshift) and one moderate-impact (missense) candidate variant were identified, including a missense variant in *DMD* and a frameshift variant in *CLIC2*. The *DMD* missense variant (c.2207T>C (p.Gln736Arg)) results in a single amino acid change from glutamine (Gln) to arginine (Arg) at amino acid 736, resulting in a non-conserved substitution (VarElect score: 183.84). Multiple sequence alignment (MSA) was performed by ClustalW [36] with default parameters; the MSA revealed that the residue corresponding to the feline variant site is highly conserved across species (Figure 5).

The p.Gln736Arg substitution within the DMD gene occurs within a spectrin repeat, which is composed of tightly packed α-helical bundles. In this structural context, residue side chains contribute to helix packing and stabilization through hydrogen bonding and electrostatic interactions. The frameshift variant in *CLIC2* (c.823-824CAG>C (p.Leu275Ter)) results in the formation of a truncated protein and loss of function (VarElect score 3.26) and is thought unlikely to be the causative variant for DD-MD in this cat.

## 4. Outcome

At the time of manuscript submission, the cat was alive (1.5 years after diagnosis), with no additional episodes of rhabdomyolysis, although tongue protrusion, cervical and thoracic limb muscle hypertrophy, and a stiff gait continue. Ongoing management for the cat is focused on a continued novel protein diet for the food-responsive enteropathy and supportive and symptomatic care for myopathy-related clinical signs. Inhalant anesthesia is avoided. Additional cardiac evaluation was not pursued.

## 5. Discussion

Here, we report a previously unrecognized variant in *DMD* that is likely causative of DD-MD in this cat. Histopathology, immunohistochemistry and WGS were utilized to identify causative variants for dystrophin deficiency. Comparison of the cat’s genome to a reference healthy cat genome identified two potential disease-causing variants that were not found in the reference cat population. The p.Gln736Arg substitution within the DMD gene occurs within a spectrin repeat, which is composed of tightly packed α-helical bundles. In this structural context, residue side chains contribute to helix packing and stabilization through hydrogen bonding and electrostatic interactions. Glutamine is a neutral polar residue with moderate side-chain length and flexible hydrogen bonding capability, whereas arginine is larger and positively charged, with an extended side chain that can introduce additional electrostatic interactions. Substitution of glutamine with arginine at this position may therefore disrupt local helix packing due to steric effects and alter the electrostatic environment within the helical bundle.

Peracute rhabdomyolysis has previously been reported in cats with DD-MD following inhalant anesthesia [3,6]. Reported cats displayed similar clinical and biochemical findings to the cat reported here, including hyperphosphatemia, hypocalcemia, and marked increases in CK activity [3]. Rhabdomylosis may occur due to abnormal sarcolemmal permeability associated with dystrophin deficiency, which further deteriorates after exposure to inhalant anesthetics [3]. Given the potential association between inhalant anesthetic agents and rhabdomyolysis in such patients, a total intravenous anesthesia technique was used for muscle biopsy collection and resulted in no known complications. It is therefore recommended to avoid inhalant anesthetic agents in cases suspected of having DD-MD.

The significance of the *CLIC2* variant is not fully understood but is unlikely to be the sole cause of the muscular dystrophy observed in this cat. The *CLIC2* (c.823-824CAG>C (p.Leu275Ter)) variant is on the X chromosome, which influences its expression, and is predicted to result in the formation of a truncated protein and loss of function. *CLIC2* variants are not associated with muscular dystrophies in humans but are instead associated with intellectual disability, cardiomegaly, and potentially neurologic complications [37,38]. Intellectual disabilities may be difficult to determine in cats with *CLIC2* variants. The cat in this report was noted to have mild generalized cardiomegaly during one visit, but a complete cardiac evaluation was not performed. Neither a murmur or arrhythmia were noted on physical examination, but an electrocardiogram or echocardiogram were not performed. Cardiomyopathy is a common finding in DD-MD, and an additive role in the development of cardiomyopathy should be investigated [39]. The low VarElect score also makes this variant unlikely to be causative compared to the score for the *DMD* variant.

Retching and ptyalism may have been related to the underlying food-responsive enteropathy but could have also been exacerbated by the MD and associated hypertrophic tongue. The diaphragm was not noted to be thickened on thoracic imaging. The dysphagia was managed through transition to a canned diet, which improved clinical signs. The prior episode of constipation may have also been a manifestation of the underlying chronic enteropathy.

This study has a few limitations. Firstly, given that the cat was adopted from a rescue, the pedigree of the cat was unknown. Additionally, the whole genome sequencing output was compared against a single healthy cat reference genome rather than many healthy cat genomes. In any case, this recently identified *DMD* variant broadens the spectrum of known variants causing DD-MD in cats.

## 6. Conclusions

Here, we reported a cat with X-linked DD-MD associated with a putative new missense variant in the *DMD* gene (c.2207T>C (p.Gln736Arg)). Clinical, histopathologic, immunohistochemical and biochemical features were all consistent with DD-MD. While an additional variant was identified in *CLIC2*, its clinical relevance to the skeletal myopathy is considered low. This report expands on previously reported DD-MD variants and emphasizes the importance of considering the disease in cats presenting with tongue protrusion, muscular hypertrophy, dysphagia, persistently and markedly elevated CK activity, or unexplained episodes of rhabdomyolysis, especially in the context of inhalant anesthesia exposure. Avoidance of inhalant anesthetics and implementation of supportive, symptom-directed management remain key aspects of care for affected cats. Further investigation into the functional consequences of this newly identified *DMD* variant, as well as broader genomic comparisons across healthy populations, will be essential to better characterize genotype–phenotype relationships in feline DD-MD.

## Data Availability

The data supporting the findings of this study are not publicly available due to clinical patient privacy and confidentiality restrictions.

## Supplementary Materials

The following supporting information can be downloaded at https://www.mdpi.com/article/10.3390/ani16081278/s1.
